# Effects of Smoking on the Teeth

**Published:** 1880-06

**Authors:** 


### ARTICLE X.

### *The Effect of Smoking on the Teeth.*

At a late meeting of the Odontological Society of Great Britain, Mr. Hepburn read a paper on this subject, and the results of his investigations on the subject are contrary to what is, we believe, the popular notion. He considers that the direct action of nicotine upon the teeth is decidedly beneficial. The alkalinity of the smoke must necessarily neutralize any acid secretion which may be present in the oral cavity, and the antiseptic property of the nicotine tends to arrest putrefactive changes in carious cavities. In addition he is inclined to believe that the dark deposit on the teeth of some habitual smokers is largely composed of the carbon with which tobacco-smoke is impregnated. It is this carbon which is deposited upon the back part of the throat and lining membrane of the bronchial tubes; and with whatever disastrous effect it may act in these situations, he thinks we are justified, from what we know of its antiseptic properties, in concluding that its action upon the teeth must be beneficial. Moreover this deposit takes place exactly in those positions where caries is most likely to arise, and on those surfaces of the teeth which escape the ordinary cleansing action of the brush. It is found interstitially in all minute depressions, and filling the fissures on the coronal surfaces. It may be removed with scaling instruments from the surface of the enamel, but where it is deposited on dentine this structure becomes impregnated and stained. Indeed it is only where the enamel is faulty and there is access to the dentine that any true discoloration of the tooth takes place; but it is remarkable, he says, how the stain will penetrate through even minute cracks, provided the necessary attention to cleanliness be not exercised. The staining power of tobacco-oil may be seen when a deposit has taken place on the porous surface of tartar collected on the posterior surface of the inferior incisors. In this situation a shiny ebony appearance is occasionally produced. That tobacco is capable of allaying, to some extent, the pain of toothache, is, he thinks, true; its effect being due not only to its narcotizing power, but also to its direct action upon the exposed nerve; and he is inclined to attribute the fact of the comparatively rare occurrence of toothache among sailors in a great measure to their habit of chewing. He has been struck, in the case of one or two confirmed smokers who have come under his notice, by the apparent tendency which exists toward the gradual production of complete necrosis of carious teeth, and the various stages of death of the pulp and death of the periosteum taking place without pain or discomfort to the patient. This condition may of course be brought about by a variety of influences, but in these special cases he is inclined to think that the presence of nicotine in the mouth has acted powerfully. The experience of other speakers in the subsequent discussion appeared to corroborate that of Mr. Hepburn, except that Mr. Oakley Coles thought the frequent changes of temperature probably injurious and tending to produce cracking of the enamel, and Mr. Arthur Underwood thought that smoking to the extent of injury to digestion tended to cause recession of the gums and otherwise to injure the nutrition of the teeth. —*British Medical Journal.*

---

